# Proceedings of the 13th annual deep brain stimulation think tank: the evolving landscape

**DOI:** 10.3389/fnhum.2026.1770451

**Published:** 2026-03-11

**Authors:** Chance R. Fleeting, Eduardo M. Moraud, Kamil Uğurbil, Doris D. Wang, Wolf-Julian Neumann, Andrea A. Kühn, Valerie Voon, Victor Pikov, Marie-Laure Welter, Michael D. Fox, John D. Rolston, Mahsa Malekmohammadi, Yagna J. Pathak, Lyndahl M. Himes, David Greene, Abbey S. Holt-Becker, Gabriel Lázaro-Muñoz, Alexander W. Charney, Amanda R. Merner, Martijn Figee, Katherine W. Scangos, Timothy Denison, Kent Leyde, Aysegul Gunduz, Helen M. Bronte-Stewart, James C. Beck, Nora Vanegas-Arroyave, Marta San Luciano, Norbert Brüggemann, Kelly D. Foote, Michael S. Okun, Joshua K. Wong

**Affiliations:** 1 Norman Fixel Institute for Neurological Diseases, University of Florida, Gainesville, FL, United States; 2 J. Crayton Pruitt Family Department of Biomedical Engineering, Herbert Wertheim College of Engineering, University of Florida, Gainesville, FL, United States; 3 Department of Clinical Neurosciences, University Hospital Lausanne (CHUV), Lausanne, Switzerland; 4 Defitech Center for Interventional Neurotherapies (NeuroRestore), University Hospital Lausanne and Ecole Polytechnique Fédérale de Lausanne, Lausanne, Switzerland; 5 Center for Magnetic Resonance Research, Radiology, Medical School, University of Minnesota Twin Cities, Minneapolis, MN, United States; 6 Department of Neurological Surgery, University of California, San Francisco, San Francisco, CA, United States; 7 Movement Disorder and Neuromodulation Unit, Department of Neurology, Charité—Universitätsmedizin Berlin, Berlin, Germany; 8 Einstein Center for Neurosciences Berlin, Charité—Universitätsmedizin Berlin, Berlin, Germany; 9 Department of Psychiatry, University of Cambridge, Cambridge, United Kingdom; 10 Institute of Science and Technology for Brain-Inspired Intelligence, Fudan University, Shanghai, China; 11 Medipace Inc., Pasadena, CA, United States; 12 Paris Brain Institute, CNRS UMR 7225, INSERM 1127, Sorbonne University, Paris, France; 13 Neurophysiology Department, CHU Rouen, Rouen University, Rouen, France; 14 Department of Neurology, Psychiatry, and Radiology, Center for Brain Circuit Therapeutics, Brigham and Women's Hospital, Harvard Medical School, Boston, MA, United States; 15 Department of Neurosurgery, Mass General Brigham, Harvard Medical School, Boston, MA, United States; 16 Department of Neurosurgery, University of California, Los Angeles, Los Angeles, CA, United States; 17 Boston Scientific Neuromodulation, Valencia, CA, United States; 18 Neuromodulation Division, Abbott, Plano, TX, United States; 19 NeuroPace, Inc., Mountain View, CA, United States; 20 Restorative Therapies Group Implantables, Research, and Core Technology, Medtronic Inc., Minneapolis, MN, United States; 21 Center for Bioethics, Harvard Medical School, Boston, MA, United States; 22 Department of Psychiatry and Behavioral Sciences, Massachusetts General Hospital, Boston, MA, United States; 23 The Windreich Department of Artificial Intelligence and Human Health, Mount Sinai Medical Center, New York, NY, United States; 24 Center for Neurological Restoration, Cleveland Clinic, Cleveland, OH, United States; 25 Nash Family Center for Advanced Circuit Therapeutics, Icahn School of Medicine at Mount Sinai, New York, NY, United States; 26 Department of Psychiatry, Icahn School of Medicine at Mount Sinai, New York, NY, United States; 27 Department of Neuroscience, Icahn School of Medicine at Mount Sinai, New York, NY, United States; 28 Department of Neurosurgery, Icahn School of Medicine at Mount Sinai, New York, NY, United States; 29 Department of Neurosurgery, Perelman School of Medicine, University of Pennsylvania, Philadelphia, PA, United States; 30 Department of Psychiatry, Perelman School of Medicine, University of Pennsylvania, Philadelphia, PA, United States; 31 Medical Research Council Brain Network Dynamics Unit, Nuffield Department of Clinical Neurosciences, University of Oxford, Oxford, United Kingdom,; 32 Department of Engineering Science, University of Oxford, Oxford, United Kingdom; 33 Cadence Neuroscience, Redmond, WA, United States; 34 Department of Neurology and Neurological Sciences, Stanford University School of Medicine, Stanford, CA, United States; 35 Department of Neurosurgery, Stanford University School of Medicine, Stanford, CA, United States,; 36 Parkinson's Foundation, New York, NY, United States; 37 Department of Neurology, Baylor College of Medicine, Houston, TX, United States; 38 Department of Neurology, University of California, San Francisco, San Francisco, CA, United States; 39 Institute of Neurogenetics, University of Lübeck, Lübeck, Germany; 40 Section for Movement Disorders, Department of Neurology, University Hospital of Schleswig-Holstein, Lübeck, Germany; 41 Department of Neurosurgery, University of Florida, Gainesville, FL, United States; 42 Department of Neurology, University of Florida, Gainesville, FL, United States

**Keywords:** closed loop stimulation, deep brain stimulation, neuroethics, neurogenetics, Neuromodulation

## Abstract

The Deep Brain Stimulation (DBS) Think Tank XIII was held September 2-4th, 2025, in Gainesville, Florida, at the Norman Fixel Institute for Neurological Diseases at the University of Florida. The theme was “The Evolving Landscape of DBS: New Indications, New Goals.” This theme was a continuation of the DBS Think Tank XI and XII, which were focused on emerging technology and pushing the horizon of indications. Since its founding in 2012, the DBS Think Tank has provided a global forum for leading clinicians, engineers, and researchers in both in industry and academia to present, discuss, and debate the current state of DBS technologies as well as to consider important logistics and ethical challenges. Over the course of three days, members of each panel presented and facilitated discussions on the cutting edge of DBS research. The keynote speaker was Dr. Kamil Uğurbil of the University of Minnesota, who led the first group of researchers to demonstrate the feasibility of imaging the human brain using fMRI technology and who was a pioneer in the development of high-field human MRI scanning. Nobel laureate Dr. Stanley Prusiner, from the University of California, San Francisco, used the story of the discovery of prions to demonstrate the power of pursuing a finding even when the idea conflicted with the prevailing state of the field. The think tank was divided into sections, including: Next Generation Neuromodulation for Gait, Brain Networks and Neuromodulation, Neuroscience & Society, Interventional Psychiatry & Behavior, Devices for Closing the Loop, Physiology & Closing the Loop, and A Roadmap for Genetics & Neuromodulation.

## Introduction

The 13th Annual Deep Brain Stimulation (DBS) Think Tank was held September 2nd-4th, 2025, at the Norman Fixel Institute for Neurological Diseases at the University of Florida in Gainesville, FL. Sessions were broadcast live via Zoom to a broad forum including researchers, clinicians, industry professionals, and individuals with neuromodulation devices. Currently, the think tank group estimates that there have been at least 300,000 DBS devices placed for neurological and neuropsychiatric conditions. Leading members in the fields of medicine, academia, engineering, and industry freely discussed and debated current and emerging advances, inclusive of logistical and ethical challenges.

Corresponding with this years theme of “The Evolving Landscape of DBS: New Indications, New Goals,” DBS Think Tank XIII focused on seven domains in addition to talks from UCSF based Nobel Laureate Dr. Stanley B. Prusiner on the discovery of prions, and a keynote address by Dr. Kamil Uğurbil of the University of Minnesota on the pioneering development of fMRI and higher field human MRI imaging. Specifically, the areas discussed this year were the following:

1. Next generation neuromodulation for gait
2. Brain networks and neuromodulation
3. Neuroscience & society
4. Interventional psychiatry & behavior
5. Devices for closing the loop
6. Physiology & closing the loop
7. A roadmap for genetics & neuromodulation

### Developing tools for studying the human brain

**Figure 1:**
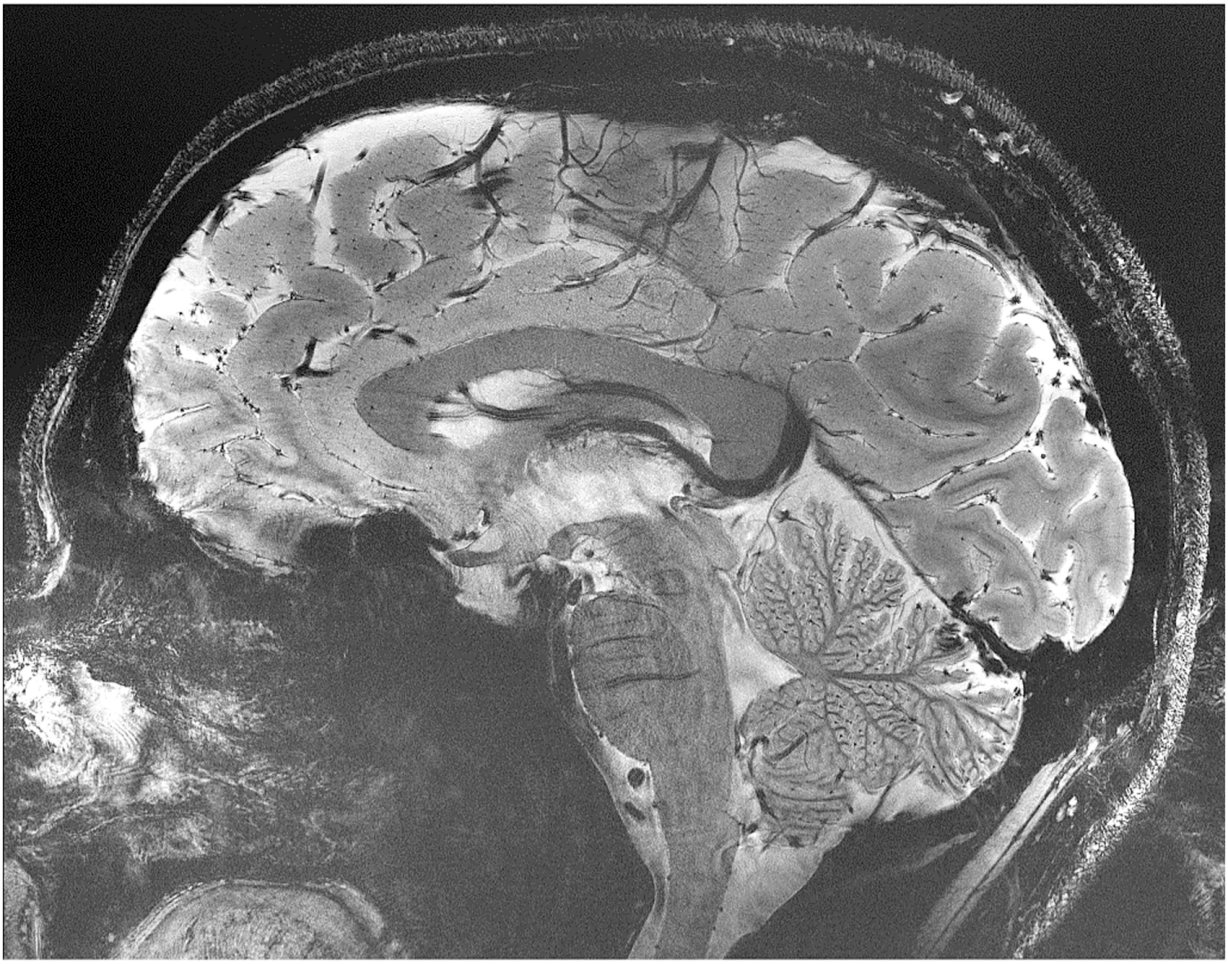
Representative gradient recalled echo image of a sagittal slice of the human brain obtained at 10.5 Tesla with 0.2 × 0.2 mm^2^ in-plane resolution and 2 mm slice thickness in slightly under 5 mins (Lagore et al., 2025).

**Figure 2:**
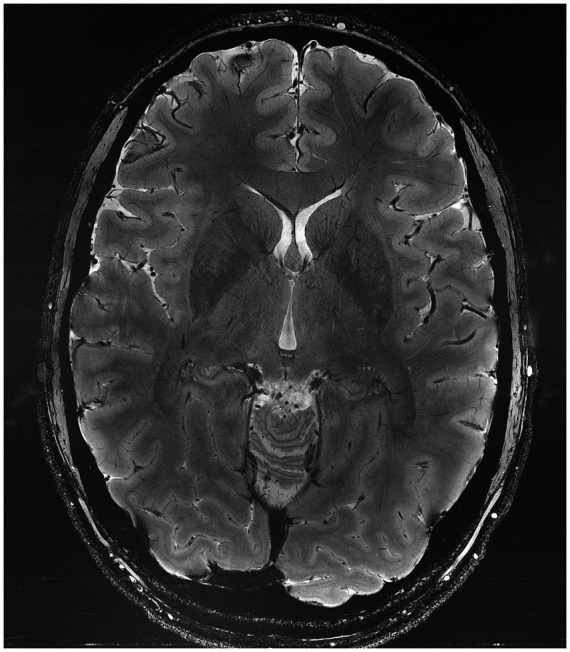
Representative gradient recalled echo image of an axial slice of the human brain obtained at 10.5 Tesla with 0.2 × 0.2 mm^2^ in-plane resolution and 2 mm slice thickness in slightly under 5 mins (Lagore et al., 2025).

Scientific advances strongly rely on the development of novel tools that enable the acquisition of previously unobtainable information. This philosophy has been the guiding principle in Dr. Uğurbil’s research center, the Center for Magnetic Resonance Research (CMRR). This pathway has proven to be critically aligned with national initiatives such as the human connectome project and the BRAIN initiative. Dr. Uğurbil’s focus has been on magnetic resonance imaging (MRI) techniques for obtaining functional, physiological, and metabolic information in the BRAIN. Cognizant of the sensitivity and resolution challenges faced, he pioneered instrumentation and techniques for human imaging at high magnetic fields. His group conducted one of two independent and simultaneous efforts to introduce functional brain imaging (fMRI), at the time using a magnetic field of 4 tesla when clinical MRI scanners operated at ≤1.5 T. He and his group subsequently developed 7 tesla to expand fMRI to the mesoscopic scale, and to improve its sensitivity, accuracy, and reproducibility at any spatial resolution (Ugurbil, 2014). Commercial adaptation followed, establishing 7 tesla as the most advanced platform for the study of the human brain (Yacoub et al., 2001). More recently, his group has been focused on pushing the ultrahigh field ceiling to above 10 tesla and demonstrating major gains in anatomical and functional imaging of the human brain at 10.5 Tesla (Uğurbil, 2021) (Figures 1, 2). MRI-based methods have achieved transformative changes in our understanding of the human brain and management of brain pathologies, contributing to the precision of our stereotactic targeting capabilities and direct visualization of cortical and subcortical brain regions (Cho et al., 2010). We anticipate that ultrahigh magnetic fields will continue to play an indispensable role in the future development of neuromodulation therapies.

## Next generation neuromodulation for gait

While stabilization and support of gait in the context of Parkinson’s disease and other movement disorders are classic and quintessential goals of DBS, they are still areas in which we strive to improve. Recent proposed advancements in neurotechnology, such as the hybridization of deep brain and spinal stimulation, have shown distinct promise in bringing us ever closer to the precipice of an ideal therapy that would allow patients to walk as they would without their underlying conditions.

### Gait-phase-synchronized adaptive pallidal DBS for Parkinson’s disease

**Figure 3:**
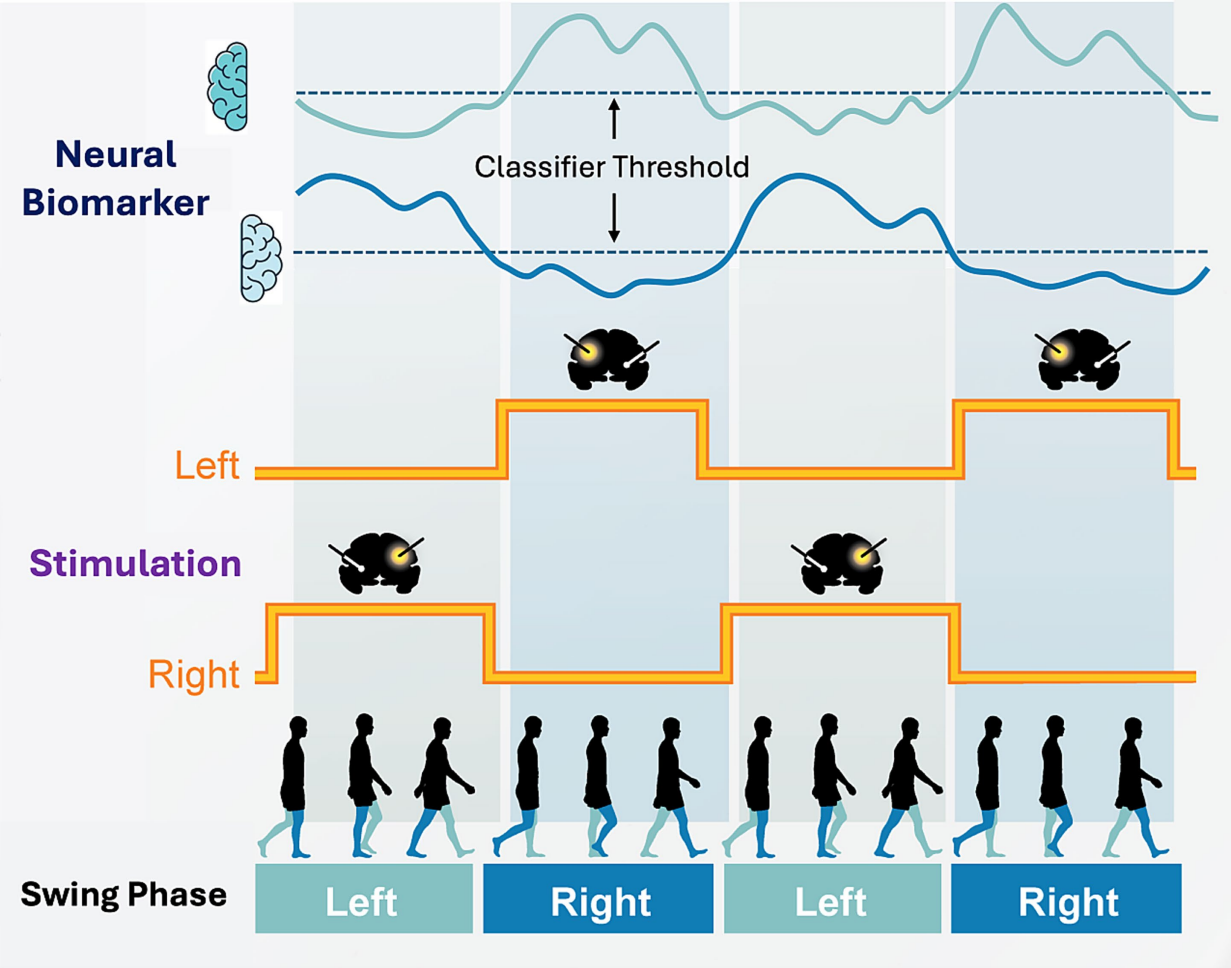
Schematic diagram of gait-phase synchronized adaptive DBS (aDBS): By using neural field potential data from the pallidum or cortical areas of left and right brain hemispheres, Dr. Doris Wang’s team identified neural biomarkers (top traces) of left and right leg swing phase (bottom panel). On-device classifier were programmed to increase stimulation amplitude during contralateral leg swing (middle traces). This method of aDBS dynamically changes the stimulation amplitude across the two brain hemispheres during natural overground walking, which may closely reflect naturalist changes that occur during the gait cycle during walking (Louie et al., 2025).

Gait dysfunction is a major source of disability in Parkinson’s disease (PD) and frequently remains refractory to conventional continuous deep brain stimulation (cDBS). Dr. Doris Wang’s team developed a neuromodulation framework that aligns stimulation with locomotor dynamics and uses data-driven programming strategies to optimize the outcome of gait (Louie et al., 2025). First, they implemented gait-phase-synchronized adaptive DBS (aDBS) that transiently modulates pallidal stimulation amplitude during contralateral leg swing using individualized cortical–pallidal biomarkers recorded by a chronically implanted bidirectional neurostimulator (Figure 3). In five PD participants, the system enabled real-time gait phase detection, reduced gait variability, and improved symmetry. In a double-blinded crossover trial comparing this approach with cDBS, aDBS was well tolerated and reduced falls without worsening global motor control (Louie et al., 2025). Second, they tested a gait-triggered aDBS paradigm that switched to a gait-optimized setting only when a subject was walking. The team developed a Walking Performance Index and applied Gaussian-process modeling to map stimulation parameters to gait outcomes, identifying personalized settings to improve walking (Fekri Azgomi et al., 2025). Finally, they established an at-home decoding pipeline by combining implant-based sensing with wearable sensors. Across more than 80 h of recordings in four PD subjects, sensitive and specific biomarkers were derived to accurately classify walking versus non-walking and the biomarkers were compatible with on-board classifiers, enabling translation to real-world, state-contingent aDBS. In a blinded short-term clinical comparison of gait-triggered aDBS versus cDBS, one participant revealed improved gait metrics and preferred aDBS (Wang D. et al., 2025). Movement-synchronized aDBS that was driven by personalized biomarkers was feasible, safe, and clinically promising. By restoring physiological timing and enabling state-contingent neuromodulation, this framework offers a potential path to more durable overall gait improvement and, possibly in the future, to balance.

#### Activity-dependent adaptive deep brain stimulation alleviates locomotor deficits in people with Parkinson’s disease

PD results in a spectrum of locomotor deficits, including gait abnormalities, postural instability, and freezing of gait, that fluctuate with daily mobility activities and medication states. Many of these impairments remain inadequately treated by conventional deep brain stimulation (DBS), which delivers continuous, activity-agnostic stimulation that is commonly optimized for tremor, rigidity, or bradykinesia (Fasano et al., 2015). As a result, millions of patients continue to experience disabling gait and balance problems that cause frequent falls, restrict independence, and increase comorbidities.

**Figure 4:**
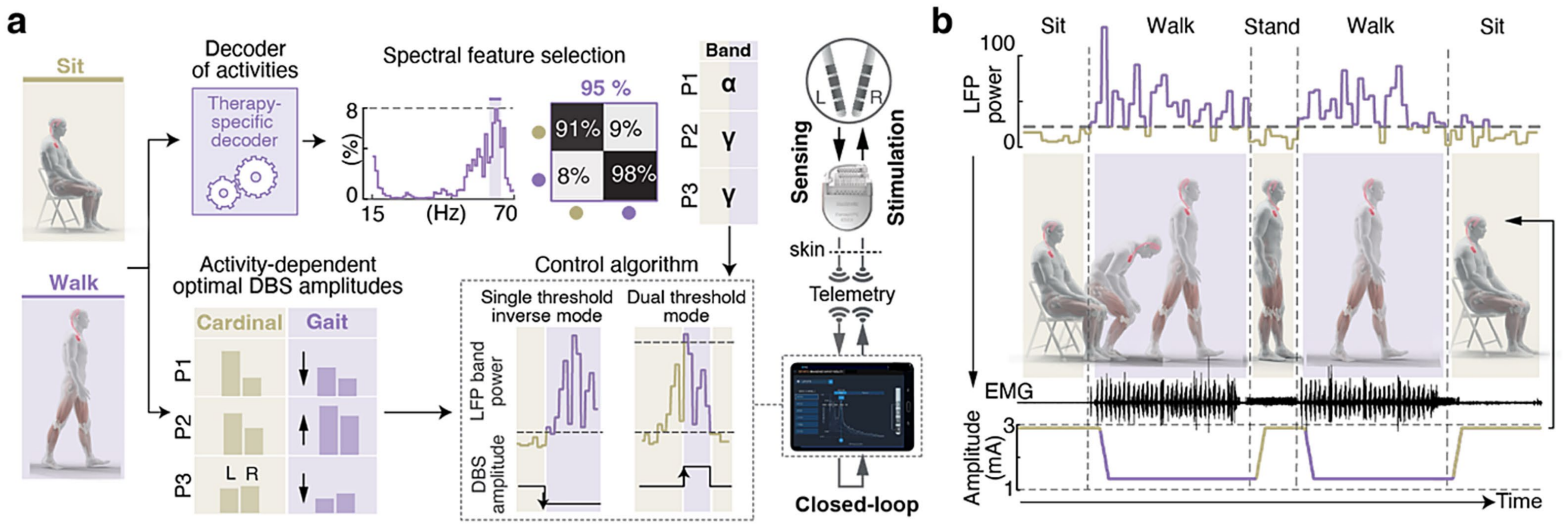
Activity-dependent adaptive DBS modulates stimulation based on gait-related neural signals. **(a)** Workflow for stated decoding: LFPs from the STN are processed to select spectral features (e.g., *α* or *γ* bands) that distinguish sitting from walking. Participant-specific (P1, P2, P3) amplitudes are determined for “Cardinal” (sitting/standing) and “Gait” (walking) states to drive the closed-loop control algorithm. **(b)** Real-time modulation showing how closed-loop control dynamically alters stimulation amplitude during sitting, standing, and walking. As LFP power increased during walking, the algorithm automatically lowers the amplitude to the optimal gait setting. Key: P1-3: Patients 1, 2, 3; STN: Subthamic Nucleus; LFP: Local Field Potentials; α band: the alpha frequency band (8–12 Hz); γ band: the gamma frequency band (>30 Hz); EMG: electromyogram.

Dr. Eduardo M. Moraud and his team introduced an activity-dependent DBS therapy that embeds distinct stimulation parameters optimized for either cardinal PD symptoms (tremor, rigidity, bradykinesia) or for gait deficits. Stimulation delivery under each set of parameters was controlled in real time by neural biomarkers of gait (Figure 4) (Thenaisie et al., 2022). By identifying the physiological principles underlying the encoding of mobility states (including sitting, standing, walking, and obstacle avoidance) in the subthalamic nucleus (STN), they achieved real-time decoding of these activities using STN local field potentials. This decoding framework enabled DBS adjustments in real time depending on whether patients were sitting, standing, or walking.

In collaboration with Medtronic, they implemented this strategy on a next-generation neurostimulation platform with advanced monitoring and control features, including band-specific up- or down-regulation of stimulation amplitude (Thenaisie et al., 2021). In a proof-of-concept study in three individuals in real-world settings, activity-dependent DBS improved both locomotor deficits and the cardinal motor symptoms experienced in PD (Scafa et al., 2025).

These results demonstrated the potential for activity-dependent DBS as a method to address the full spectrum of PD motor impairments, despite frequent heterogeneous clinical profiles. Future platforms should extend beyond amplitude modulation to also develop and adjust stimulation frequency and contact configuration, thereby enabling the real time selective targeting of gait-related pathways (Horn and Neumann, 2025). The decoding framework derived by their team provides a blueprint for next-generation neuromodulation therapies that dynamically adapt stimulation to the behavioral context, to the mobility demands, and to ongoing physiology.

### Combining anatomical targeting with adaptive stimulation for gait in Parkinson’s disease

Gait and balance disorders, and particularly freezing of gait (FOG), remain among the most disabling and treatment-resistant symptoms in PD. While subthalamic deep brain stimulation (STN-DBS) robustly improves tremor, rigidity, and bradykinesia, its effect on FOG is highly variable. Up to one-third of patients experience persistent or even new-onset FOG after STN-DBS surgery. Recent advances in anatomical and physiological mapping have identified “sweet spots” for stimulation within the STN region. Directional DBS targeting the central STN improves gait initiation and postural control more effectively than posterior or conventional ring-mode stimulation, with preferential recruitment of premotor and mesencephalic locomotor pathways. Complementary electrophysiological recordings have revealed distinct gait initiation states associated with absence of FOG, predisposition, or imminent occurrence. These have been linked to low- and high-beta activity distributed differently across central versus posterior STN subregions. These insights could pave the way for adaptive DBS (aDBS) strategies aimed to tailor stimulation to the patient’s neural and motor state. Early clinical trials of beta-driven aDBS highlight the importance of parameter selection and controllability as a method for effective suppression of pathological beta activity, while chronic feasibility studies demonstrate superior control of residual motor symptoms when compared to continuous DBS that has been fully optimized. Beyond beta activity, stimulation-entrained gamma oscillations and gait-phase–locked biomarkers recorded from cortical and subcortical sites have recently emerged as promising control signals. Novel aDBS paradigms, such as beta burst driven stimulation for FOG or gait-phase–synchronized adaptive neuromodulation could potentially provide improvements in gait variability, symmetry, and in fall reduction. Together, these findings suggest that combining anatomical targeting of the central STN with physiological, state-dependent adaptive stimulation may provide a powerful framework to improve disabling gait disorders in PD. Ongoing technological developments and large-scale collaborative trials will be critical to validate personalized neuromodulation strategies and to translate them into long-term clinical benefit.

## Brain networks and neuromodulation

Ongoing advancements in connectomics and circuit-based brain mapping are redefining how DBS influences the distributed systems throughout the brain, helping us better understand its effect, both therapeutic and adverse, and thus guiding us towards more refined and precise interventions. Increasingly more evidence demonstrates that the effects of neuromodulation depend not only on stimulation location, but also in large part by patient specific factors and timing of stimulation. Together with our growing understanding of stimulation effects outside of the local networks, these insights mark a transition from spatially guided direct local stimulation to network- and state-informed approaches to DBS.

### Brain networks and neuromodulation: going from where to who and when

Recent advances have facilitated the identification of the location of brain circuits responsible for many DBS-induced changes in neurological and psychiatric symptoms. These methods combine the precise location of stimulation with maps of anatomical or functional connectivity to identify connections that co-vary with symptom change. These approaches have proven successful and have been important in identifying brain circuits responsible for improvement in individual and frequently also in co-morbid symptoms. However, as the field of “connectomic DBS” has advanced, it has become clear that circuit topography alone provides an incomplete picture. There are now examples where electrodes connected to the same brain circuit result in opposite symptomatic effects. For example, DBS sites connected to the hippocampus and an extended memory circuit have been associated with cognitive decline in PD, but also with cognitive improvement in Alzheimer’s disease (AD). To resolve this paradox, Dr. Michael Fox and his team studied electrode locations and cognitive outcomes in patients who received STN DBS for PD (2 datasets: *n* = 33, *n* = 28) or fornix DBS for AD (1 dataset: *n* = 46). DBS site connectivity to the same brain circuit, as defined by diffusion tractography for structural and resting-state fMRI for functional connectivity, was cognitively deleterious in PD but was beneficial in AD. The opposite findings were driven by patient age. In patients below 65, connectivity to the memory circuit resulted in cognitive decline. In contrast, in patients above 65, connectivity to the memory circuit was associated with cognitive improvement. These age-dependent effects were independent of diagnosis and were present in both PD and AD. Similar preliminary results have been demonstrated in DBS for depression. DBS sites connected to the depression circuit revealed opposite effects on depression symptoms depending on baseline depression severity. In patients with no baseline depression, DBS sites connected to the depression circuit resulted in depression. In contrast, in patients with high baseline depression, connectivity to the same circuit was associated with depression improvement. Together, these results suggest that the cognitive effects of DBS were not just dependent on “Where,” but also “Who,” and “When.” Additionally, age and baseline symptom severity likely play critical and underappreciated roles.

### Deep brain stimulation for disorders of consciousness: where, when, and who

DBS is emerging as a potential therapy for disorders of consciousness (DoC), conditions lacking effective interventions. In a recent *Nature Communications* study, Dr. John Rolston and the Mapping & Engineering Neural Dynamics (MEND) lab analyzed DBS in the thalamic centromedian–parafascicular complex in 40 patients (Warren et al., 2025). Clinical improvements were linked to stimulation extending into the inferior parafascicular nucleus and ventral tegmental tracts, underscoring that *where* stimulation engages specific subcortical circuits seemed to be a key to recovery.

Equally important was *when* stimulation was delivered. Lessons from responsive neurostimulation in epilepsy revealed that timing relative to brain state influenced long-term outcome(s) (Anderson et al., 2024). Aligning stimulation with optimal physiological windows has the potential to promote plasticity more effectively than continuous or untuned approaches.

Finally, *who* receives DBS mattered. Not all patients responded equally, and in DoC, the preserved striatal and cortical integrity predicted better outcomes. Individual connectivity and neural reserve likely defined responsiveness, suggesting the need for precision in patient selection.

Together, these findings suggested that DBS efficacy was dependent on more than electrode location. Considering the *where, when,* and *who* of stimulation can potentially guide a more personalized, mechanistically grounded approach to neuromodulation in DoC and beyond.

### Fusing connectomics and neurophysiology for network discovery and adaptive circuit targeting in Parkinson’s disease

**Figure 5:**
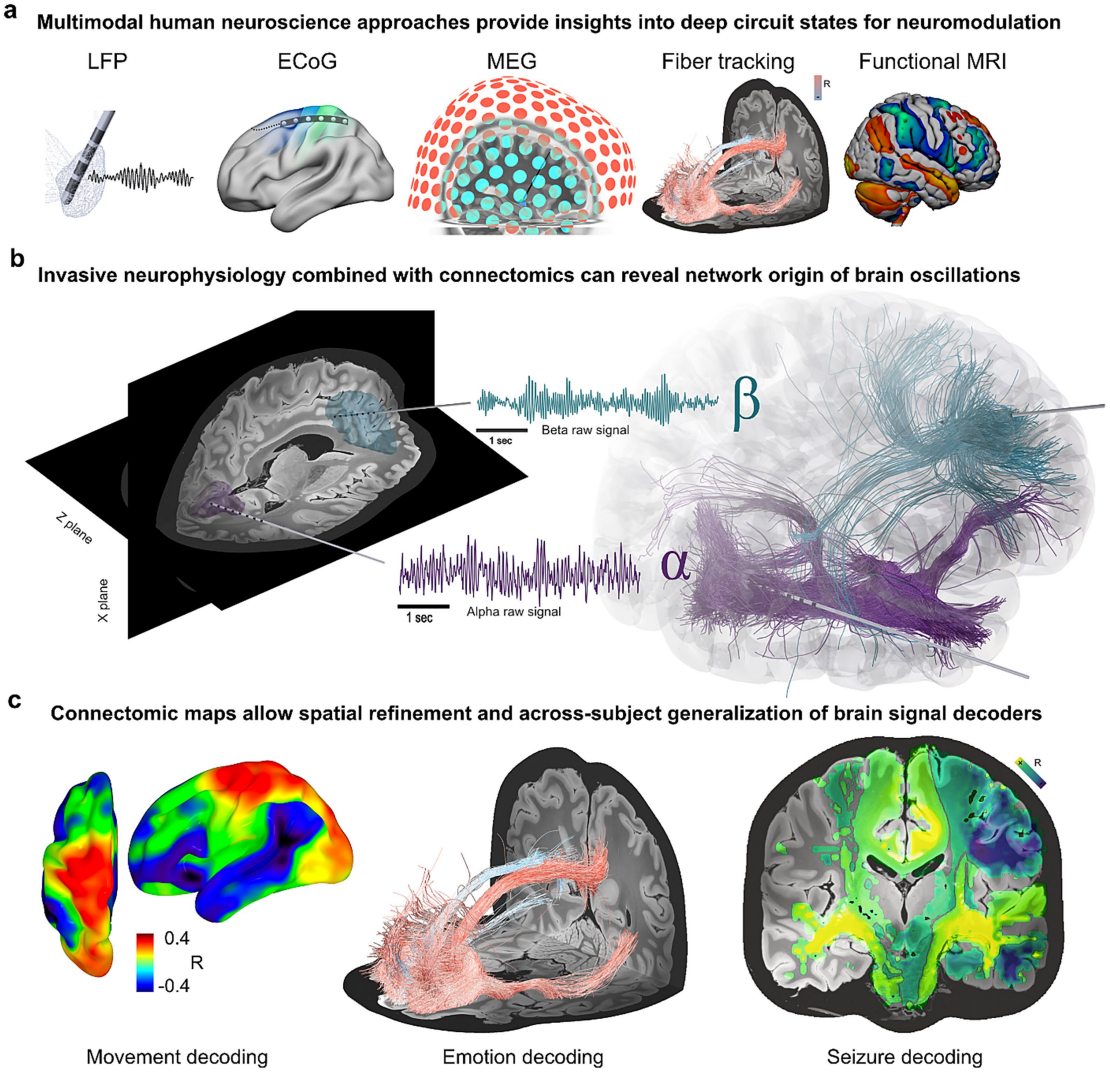
Multimodal and connectomic approaches for decoding and targeting deep brain circuits. **(a)** Multimodal human neuroscience methods, including invasive and non-invasive recordings together with structural and functional imaging, provide complementary access to deep circuit dynamics relevant for neuromodulation. **(b)** Invasive neurophysiology combined with connectomic mapping reveals the network origin of brain oscillations, dissociating α- and *β*-band activity within large-scale cortico-subcortical pathways [adapted from Chikermane et al. (2024)]. **(c)** Connectomic maps support spatial refinement and across-subject generalization of brain signal decoders. This enables transfer learning for diverse domains such as movement, emotion, and seizure decoding, providing anatomical validation for cross-circuit decoding and non-motor networks [adapted from Merk et al. (2025)].

Advances in DBS, such as those shown in Figure 5, increasingly combine invasive neurophysiology with connectomics to move beyond empirical stimulation and toward network-based interventions. Merging these modalities reveals neural dynamics within large-scale brain networks, such as stopping circuits and dopamine-related *β*-band activity. Connectomic analyses can separate pathway-specific oscillatory components (e.g., high-β hyperdirect, low-β indirect) and capture network effects even from transient, state-dependent closed-loop stimulation, where supplementary motor and striatal activity can reinforce behavioral states.

Connectomic circuit maps can also enable transfer learning across individuals, supporting out-of-the-box decoding of movement-responsive signals. These circuit maps provide anatomical validation for cross-circuit decoding. For example, hyperdirect pathway connectivity will predict decoding performance from cortical to subthalamic sites and can identify circuits underlying non-motor domains such as emotion and memory.

Dr. Wolf-Julian Neumann’s team proposes adaptive circuit targeting as a framework that unites symptom decoders with real-time network steering (Merk et al., 2025). While not yet clinically feasible, this approach could leverage additional Electrocorticography (ECoG) coverage to improve decoding without requiring subject-specific parameter tuning, while remaining robust for differentiating physiological and technical artifacts. Using multivariate and multisite recordings, this method has the potential to predict motor and non-motor symptoms as well as to enable stimulation of relevant networks and to do so precisely when symptoms arise.

By embedding physiological decoding within anatomically grounded network maps, adaptive circuit targeting represents a conceptual shift toward state-specific, network-informed neuromodulation. This approach outlines a pathway toward next-generation DBS systems capable of both decoding brain states and dynamically steering stimulation across distributed circuits.

## Recent advances in neuromodulation technologies from the industry sector

The Industry Blitz is an open discussion to highlight new therapies, discuss challenges, and identify new opportunities to translate academic development into products for clinical use. This collaboration between industry and academia is a key feature essential to the DBS Think Tank.

### Boston Scientific

Boston Scientific Neuromodulation is advancing DBS through the development of innovative technologies like image-guided programming, and novel stimulation paradigms, with the overarching objective to increase efficiency and to optimize therapeutic outcomes.

The Vercise^™^ Cartesia^™^ X and HX 16-contact directional leads expand the electrode span and provide multiple levels of directionality along a single trajectory, enabling more precise engagement of targeted neural populations while reducing unintended current spread. These refinements are designed to increase therapeutic range and to decrease the likelihood of stimulation-induced adverse effects.

Programming has been further enhanced through image-guided programming (IGP). Clinical investigations demonstrate that IGP significantly reduces programming duration, while maintaining stable stimulation fields across follow-up intervals. This stability translates to sustained motor improvement and quality-of-life benefits (Lange et al., 2021; Torres et al., 2024; Aldred et al., 2023). Illumina 3D^™^ (I3D), a predictive programming algorithm, builds on this foundation by automatically identifying stimulation parameter sets that are engineered to maximize therapeutic target coverage while minimizing activation of side-effect regions, providing efficient and clinically effective starting points for parameter exploration (Malekmohammadi et al., 2022).

To further expand the therapeutic landscape, the CHRONOS research platform enables systematic investigation of temporal pattern stimulation. By supporting pulse-by-pulse composition, bursting, cycling, and flexible scheduling, among other capabilities, CHRONOS allows investigators to “speak differently” to neural circuits. These spatiotemporal paradigms aim to mitigate habituation, enhance cell- and pathway-selectivity, and potentially engage mechanisms of plasticity not accessible through tonic stimulation. Collaborative research studies are underway to assess whether these approaches can provide durable symptom relief in Parkinson’s disease, reduce therapy loss in essential tremor, and extend DBS to new indications (Kariv et al., 2024).

Together, these innovations highlight a translational pathway from engineering and algorithm development to clinically meaningful improvements, underscoring a commitment to personalized and durable neuromodulation therapies.

### Abbott

Despite ongoing innovation, the field of neuromodulation continues to face critical challenges in accessibility, patient awareness, and engagement. Previous insights reaffirm the need for digital health technologies that are simple, scalable, and tailored to bridge these gaps and ultimately enable more meaningful therapies for patients (Forum on Neuroscience and Nervous System Disorders et al., 2024). Innovations in foundational platforms such as Neurosphere^™^ digital health and the LibertaRC^™^ DBS system are central to a patient-centric innovation strategy.

Recent clinical studies collectively demonstrate the feasibility, effectiveness, and scalability of these digital platforms in DBS therapy:

1. Remote Optimization in Parkinson’s Disease (ROAM-DBS): A multicenter randomized controlled trial showed that remote programming via the Neurosphere^™^ Virtual Clinic led to significantly faster symptom improvement (15.1 days earlier on PGIC scale) compared to in-clinic programming. The study also demonstrated high patient acceptance (96%) and ease of use (84%), with no increase in adverse events, supporting the safety and utility of decentralized care models (Gharabaghi et al., 2025).
2. Feasibility of Remote Therapeutic Monitoring: A pilot study integrating consumer-grade wearables with a remote monitoring app achieved high compliance rates (up to 85.5% for monthly surveys), validating the potential for scalable, real-world data collection. This architecture lays the groundwork for a unified digital ecosystem capable of supporting personalized therapy adjustments and longitudinal tracking (Thankathuraipandian et al., 2024).
3. DBS for Treatment-Resistant Depression (TRD): A pooled analysis of 172 patients implanted with subcallosal cingulate DBS demonstrated sustained and clinically meaningful improvements in depressive symptoms over a 5-year period. These findings support the expansion of DBS indications into neuropsychiatric populations and inform ongoing prospective trials (Himes et al., 2025).

Together, these studies underscore the importance of building layered innovation through foundational digital infrastructure, while incrementally enhancing therapeutic capabilities.

### NeuroPace

The NeuroPace Post-Approval Study (NCT0240384) is the largest FDA-reviewed prospective neuromodulation study of safety and effectiveness in drug-resistant focal epilepsy (NeuroPace, n.d.). There were 324 patients enrolled at 32 centers in the USA. Patients had a median baseline disabling seizure frequency of 6 per month. Long-term median seizure reduction was 82% at 3 years, with 42% of patients experiencing 6 + months of seizure freedom. Over the 36-month follow-up, 85% of participants who had at least 6 months of data had no generalized tonic clonic seizures (GTCs) for 6 months or more, while 62% of participants who had at least 12 months of data had no GTCs for 12 months or more.

The NAUTILUS Study (NCT05147571) is a pivotal clinical study to determine if the RNS System is safe and effective as an adjunctive therapy for the treatment of primary generalized seizures in individuals 12 years and older who have drug-resistant idiopathic generalized epilepsy (IGE). This prospective, multicenter, single-blind, randomized, sham stimulation controlled study has enrolled 100 participants within the United States. Leads were placed bilaterally in the centromedian nuclei of the thalamus. Primary outcome measures are the 12-week post-operative serious device-related adverse event rate and the time to second GTC seizure. Secondary endpoints included median percent change in days with any type of generalized seizure, days with GTCs and numbers of GTCs by time of stimulation.

The RNS System Lennox–Gastaut Syndrome (LGS) Feasibility Study (NCT05339126) is an NINDS-funded Brain Initiative study intended to generate preliminary safety and effectiveness data for brain-responsive neurostimulation of thalamocortical networks as an adjunctive therapy for reducing the frequency of generalized seizures in patients 12 years or older with LGS who are refractory to antiseizure medications. The study has implanted all 20 subjects. Leads are placed bilaterally in pre-frontal cortex and centromedian thalamic nuclei.

NeuroPace research has validated a vision transformer deep learning model to sort chronic ECOG data that will be used to assist physicians in the review of ECOG data. The model has recently demonstrated an accuracy of 96% on a separate clinician-annotated test dataset (Afzal et al., n.d.).

### Medtronic

Whereas DBS therapy has documented safety and effectiveness across multiple approved indications, optimization and personalization often still rely on trial-and-error programming by clinicians and manual adjustments by patients. Recent neurotechnology advancements that enable chronic neural sensing provide novel insight into patient-specific biofeedback that can efficiently guide where, when, and how to deliver stimulation. Commercial DBS devices with neural sensing, such as the Medtronic Percept^™^ platform embedded with BrainSense^™^ technology, enable simultaneous stimulation and chronic sensing from implanted DBS leads both in and out of clinic and have now been implanted in tens of thousands of patients worldwide. These types of next-generation DBS platforms have enabled recent innovative features aimed at improving both the clinician programming and patient therapy experiences. Furthermore, chronic neural sensing at scale will drive real-world and data-driven insights to inform future therapy and technology innovations.

There is growing evidence that neural sensing can be used for a more efficient initial DBS programming workflow (Binder et al., 2023; Duffy et al., 2023) and enhanced optimization (Baker et al., 2024). However, as these data-driven insights continue to advance our understanding of the clinical value of neural sensing applications, there is also a critical need to co-develop programming interfaces aimed at ease-of-use and automation in order to facilitate efficient and consistent identification of insights, and ultimately scale adoption. The BrainSense Electrode Identifier feature provides an example of a software update to the Percept DBS platform that automatically identifies the localization of patient-specific brain signals of interest to inform the clinician’s choice for stimulation location, thereby providing significant time savings in a DBS programming study (Thompson et al., 2025).

Neural sensing may also be applied toward automating when and how DBS therapy is delivered. For example, the Medtronic-sponsored ADAPT PD trial evaluated the safety and effectiveness of long-term aDBS in Parkinson’s disease, whereby stimulation amplitude was automatically adjusted in a personalized way to each participant’s brain signals (Stanslaski et al., 2024). The results of the ADAPT PD trial suggest that aDBS may not only alleviate patient burden from manual adjustments but also enable clinical improvements above cDBS (Bronte-Stewart et al., 2025). While aDBS for Parkinson’s disease is now commercially approved, there is a need for broader exploration into simplified workflows, patient-specific benefits, and opportunities to automate setup over long-term patient management (Busch et al., 2025). As automated features continue to be developed across DBS therapies, Percept is an example hardware platform that has been designed to be software-upgradable.

With the expanded availability of chronic neural sensing beyond investigational research environments, a significantly broader group of clinical and scientific users has relatively easy access to more data than ever before for supporting the chronic monitoring of patient states. Data access provides an opportunity to enable new therapy development and exploration in both approved and expanding indications. Insights into circadian rhythms (van Rheede et al., 2022; Cagle et al., 2024), new symptom biomarkers, or response tracking (Alagapan et al., 2023; Shute et al., 2016; Gregg et al., 2021; Provenza et al., 2024) have already been identified in chronic neural sensing data across movement, neuropsychiatric, and epilepsy disorders. With data access and identification of novel patterns, it will be critical to link these data-driven insights to clinical impact; derive simple methods to combine multiple data modalities, such as imaging and neural sensing; and develop user interfaces that make insights interpretable and accessible to all levels of users. Moreover, the explosion of Neuro AI as a powerful tool for analyzing data at scale should be applied to identify data-driven insights that focus on clinically relevant challenges or barriers to therapy optimization or adoption. Focusing on these clinically meaningful insights using neural data will be a key enabler in continued biomarker discovery across indications, automation to drive workflow efficiencies, classifier development to monitor patient states, and understanding potential mechanisms of action needed for new therapy development.

## Neuroscience and society

As neurotechnology and neurological implants have become more commonplace and integrated into public consciousness, there is growing awareness and recognition of new ethical issues and considerations. Challenges in privacy, safety, and consent have all surfaced as topics that need to be prospectively addressed.

### Defining post-trial responsibility for investigational neural devices

A major challenge in neural device development is what to do when an investigational neural device provides benefit for an otherwise treatment-resistant condition or functional impairment (Lázaro-Muñoz et al., 2018). When trials for DBS and implantable brain-computer interface (BCI) systems end, participants who benefit from these investigational devices may not have a way to ensure their maintenance. Health insurance providers can deny coverage for maintenance of these devices because they are still investigational (Hendriks et al., 2019). Device developers and manufacturers may decide to stop producing compatible hardware or necessary software, or they may go out of business (Muñoz et al., 2020). Thus, when these trials end, participants face the possibility that if the device is damaged or requires a new battery, they may not be able to access or pay for its maintenance (Okun et al., 2024). This circumstance will likely lead to deprivation harms, patients who do not have a functioning device will likely re-experience the severe treatment-resistant condition or lose functions that had been previously restored. Deprivation harms may render certain groups involved in these trials potentially liable for negligence (Fins et al., 2024). A question that would determine liability is whether the groups involved in the trials had a duty to provide maintenance. In other words, is the omission or lack of maintenance for patients who benefit from the device “unreasonably unsafe?” Dr. Gabriel Lazaro-Munoz’s group examined this issue and provided preliminary data from a nationally representative study examining public attitudes towards post-trial issues and found that 77% of respondents disagree with participants being solely responsible for the maintenance of beneficial investigational devices following the completion of a trial (Lázaro-Muñoz et al., 2022).

### The living brain project

Many neuroscience studies have historically relied on postmortem brain tissue to gain insight into human biology. While these studies have provided valuable information about anatomy and cellular composition, postmortem tissue may not fully capture the molecular and physiological processes that occur while the patient is living. Currently available tissue may not capture human thought, emotion, behavior, movement, learning, and memory. To fully understand these functions and what happens in disease states, Dr. Alexander Charney’s research team at Mount Sinai endeavored to study living human brain tissue.

To this end, the Living Brain Project was created to establish methods and collect tissue from living patients. In collaboration with neurosurgeons, neuroscientists, and patients, the project developed, tested, and applied methods to study intraoperative brain tissue collection from living patients, during DBS or other neurosurgical procedures that were performed to treat human disease. The team observed that during certain types of procedures at Mount Sinai, small amounts of tissue were being removed as part of standard clinical care and generally discarded. Instead of being discarded, the methods developed via the Living Brain Project allow such tissue to be collected and analyzed.

This work provided an opportunity to investigate *in vivo* gene expression, neuronal communication, and circuit-level processes in human tissue under physiological conditions. By integrating the collected data with clinical information, the Living Brain Project aims to advance the understanding of both normal brain function and neurological disease in a context not otherwise available.

### Rethinking success: patient-centered outcomes in deep brain stimulation

Developing patient-centered outcome measures is critical to ensuring clinicians, researchers, device developers, and future patients are aware of the full range of benefits experienced by patients who may receive DBS. A recent study examining patients’ perceptions of changes to their most valued personal characteristics following DBS for PD revealed that DBS was restorative to personally meaningful aspects of patients’ identity in addition to providing significant improvements in motor function. These findings aligned with previously published research (Merner et al., 2024; Merner et al., 2021). Importantly, the changes reported in these studies are not adequately captured by standard outcome measures, and this highlights the need for metrics to prioritize patients’ perspectives and their lived experience. Capturing these benefits has the potential to decrease concerns and stigma about DBS, and can help to facilitate better technology adoption (Merner and Kubu, 2023). A recent survey of the U.S. public revealed that, compared to other neuromodulation approaches, DBS was viewed as the most invasive and risky option. It was also considered the least acceptable option, and the least likely to be personally used by members of the public who may possibly need DBS in the future (Merner and Kubu, 2023). These data suggest that, despite its known efficacy as a treatment for PD and other neurological conditions, individuals who may need to use this technology may be unwilling to do so. By highlighting the full range of benefits experienced by patients who have received DBS and by prioritizing their perspectives, we can improve the public’s views on this technology, and we can encourage future patients to engage with DBS as a potential treatment option.

## Interventional psychiatry and behavior

DBS is emerging as a powerful tool to treat refractory psychiatric and neurobehavioral disorders. Imaging tractography, intracranial electrophysiology, and neural based signatures for symptoms and diseases have been driving the emerging field of interventional psychiatry and behavior. Adaptive closed-loop stimulation continues to emerge as a possible therapeutic application.

### Precision circuit-based targeting for depression and obsessive-compulsive disorder

Effective DBS for psychiatric conditions depends heavily on the precision targeting of symptom-specific brain circuits. The successful evolution of precision targeting in subcallosal (SCC) DBS for depression, using individualized tractography maps of previous responders, has improved long-term response rates from on average ~50% to ~80% (Figee et al., 2022). A precision DBS method applied for depression is currently being tested in a multicenter FDA breakthrough trial (TRANSCEND NCT06423430). In DBS for refractory obsessive-compulsive disorder (OCD) when targeting the anterior limb of the internal capsule (ALIC), the response rates still vary between 45–65%, possibly due to variable target locations within heterogeneous ALIC network connectivity. Dr. Figee and his team used probabilistic tractography and stimulation models from six OCD DBS responders to identify a map of specific therapeutic ALIC connections between vlPFC, vmPFC, OFC, thalamus, pallidum, brainstem and the STN (Segura-Amil et al., n.d.). When this tractography responder map was used prospectively in 18 refractory OCD patients, based on their individual high-definition diffusion scans, 15 patients (80%) successfully responded to DBS. Treatment outcomes using precision targeting were highly OCD-specific, and stimulation of lateralized prefrontal (right vmPFC, left vlPFC) and bilateral subcortical connections selectively correlated with the OCD symptom improvement. Effective high frequency stimulation (130 Hz, 90us, avg. 6 mA) was mostly confined to the planned tractography-defined contact and required minimal post-hoc adjustments, suggesting precision targeting could also possibly ease and standardize parameter programming and impact the overall clinical utilization of OCD DBS. Precision targeting is also under investigation for other diseases and symptoms potentially addressable by DBS. Preliminary results from a collaboration between Johns Hopkins and Dr. Figee’s Sinai group suggest that DBS can also improve refractory auditory hallucinations and the negative symptoms of schizophrenia when the stimulated substantia nigra reticulata (SNr) region is connected to the superior temporal gyrus and ALIC-vmPFC connections (Cascella et al., 2021). Dr. Figee’s group also found that stimulation of similar ALIC-vmPFC connections were involved in apathy improvement following STN DBS for Parkinson’s disease (De Bruin et al., 2025), suggesting that these connections may be a future target for patients with impairments in goal-directed behaviors and possibly may in the future may be applied across multiple diagnoses.

### Intracranial biomarkers and closed-loop stimulation in mood and decision-making disorders

**Figure 6:**
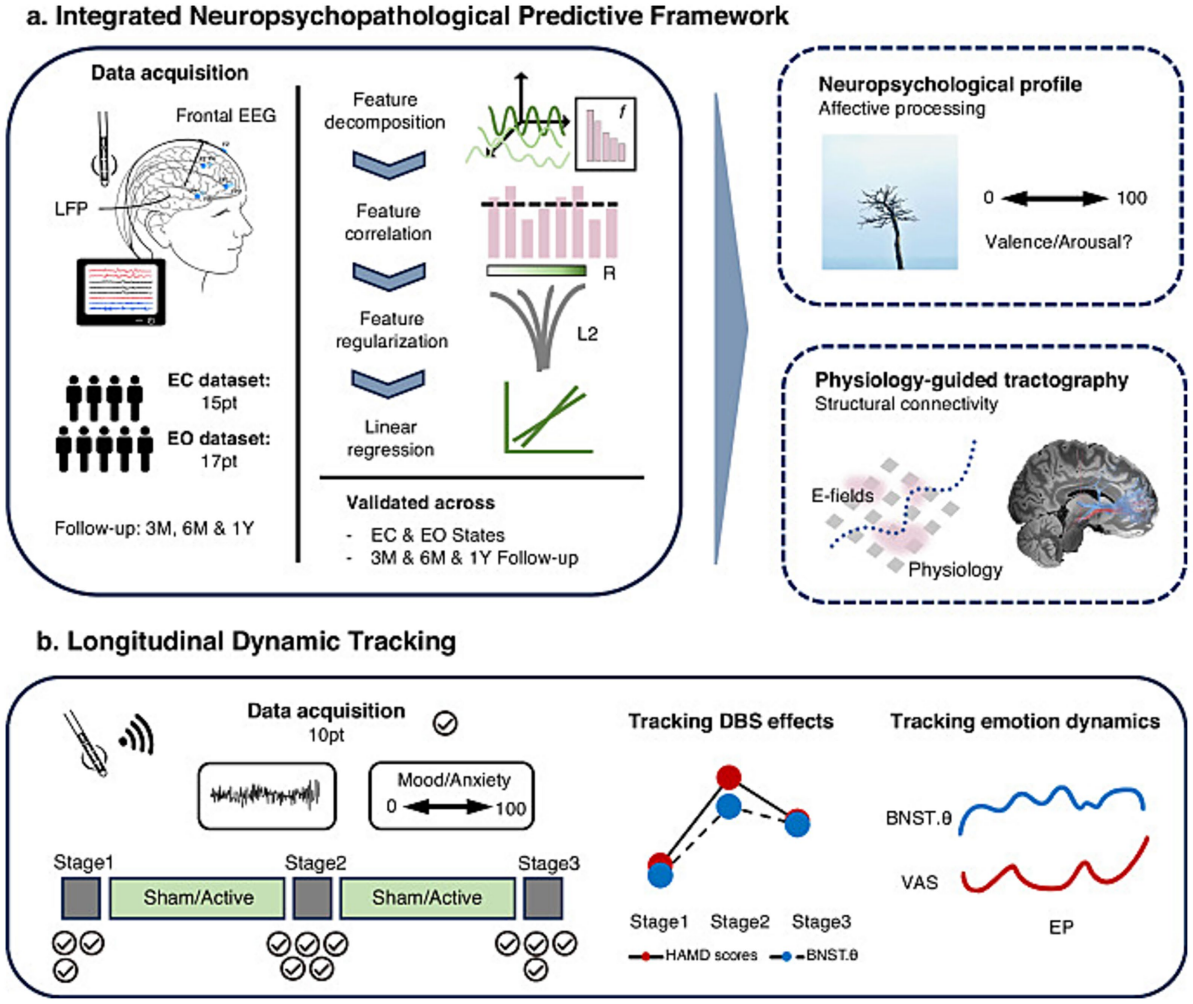
**(a)** An outline of the integrated neuropsychological predictive framework experimental design, and **(b)** an outline of the longitudinal dynamic tracking of DBS effect experimental design.

The capacity to record from DBS contacts facilitates unique insights into precise deep signals collected from local field potentials aimed at developing more objective biomarkers to guide neurostimulation. The Voon laboratory conducted a set of studies with collaborators at Ruijin Hospital focusing on biomarkers recorded at rest and task-based to enable prediction of DBS treatment outcomes, and to track transient momentary emotional states. Additionally, these biomarkers were explored for their potential for closed loop stimulation. In one study by Dr. Valerie Voon’s team, 26 patients with refractory depression underwent DBS targeting the bed nucleus of the stria terminalis (BNST) and nucleus accumbens. There was a significant clinical improvement in the open label phase with improvement of depression, anxiety, quality of life and disability. BNST theta power, prefrontal cortical theta power and prefrontal-BNST theta connectivity collected during the baseline externalization phase (DBS leads left externalized post-operatively for experimentation) predicted treatment outcomes at 3, 6 and 12 months (Wang et al., 2025b; Sonkusare et al., 2025) (Figure 6). BNST theta power during the wireless recording phase that was conducted during the randomized controlled trial was associated with depression symptoms and also tracked transient anxiety states. Chronic BNST DBS was shown to further decreased theta power.

In a second study, they ran a risk-taking card game task in PD patients recording from the STN and prefrontal cortex and this paradigm showed dissociable frequencies associated with objective parameters of risk with an increasing likelihood of loss and uncertainty. Furthermore, a separate frequency band in the STN was associated with the likelihood of betting. Open loop STN stimulation using an acute one second DBS time-locked to the risk taking decision phase paradigm decreased betting behaviors and enhanced STN theta activity (Voon et al., 2024). This theta activity during the decision phase predicted risk taking choices. Using STN theta power as a biomarker, they developed a closed-loop stimulation algorithm with one second of 130 Hz stimulation triggered when theta crossed a threshold of ~2 standard deviations above a resting baseline collected during a card game task. In nine patients, this closed loop theta-guided stimulation resulted in greater risk aversion relative to no stimulation (Wang et al., 2025a). Collectively, these studies highlight the role of intracranial physiology as potential predictive biomarkers for clinical outcomes, for tracking momentary states and as potential closed loop biomarkers.

### iEEG-guided DBS: electrophysiological biomarkers to advance targeting and enable closed-loop DBS

DBS is a promising option for patients with OCD who have not responded to standard therapy (Kayser, 2020; DuPont et al., 1995; Gadot et al., 2022), however optimal DBS targeting remains uncertain, and innovations are needed to address non-response and residual symptoms. An intracranial mapping stage utilized prior to implantation of a chronic DBS device offers an opportunity to personalize brain targets and to identify electrophysiological biomarkers of the acute clinical response to electrical stimulation (Gabrielson et al., 2021; Scangos et al., 2021). In an ongoing clinical trial (NCT05623306) of intracranial EEG (iEEG)-guided DBS for OCD, up to 15 iEEG electrodes were surgically implanted within the corticostriatal circuitry in 12 patients with OCD and they were followed by a randomized controlled study of multi-lead DBS. Results from the first four subjects revealed that stimulation across the ventral capsule/ventral striatum (VC/VS) and its regions in close proximity, the anterior limb of the internal capsule (ALIC), the dorsal anterior cingulate cortex (dACC), the STN and orbitofrontal cortex (OFC) leads to acute reproducible clinical responses. The strongest and most consistent response differed across participants, supporting the value of a personalized approach. Continuous EEG recordings were paired with multi-day clinical ratings of OCD symptoms. A machine learning model applied to spectral power features within six standard bands across the network discriminated between states of severe symptoms. Biomarkers were unique to each participant, however several features were conserved, including high-frequency activity in the OFC and frontal pole, and low-frequency activity in the STN. Connectivity across the network was mapped by applying single pulses of stimulation to each electrode contact and by examining the evoked responses in distal contacts. The strength of effective connectivity within two distinct sub-circuits was strongly correlated with the acute clinical response to stimulation. These results establish the feasibility and methodology for the identification of electrophysiological spectral power and connectivity biomarkers to guide personalized DBS target selection and to develop novel treatment paradigms such as closed-loop DBS.

## Devices for closing the loop

As neuromodulation shifts from continuous open loop stimulation to truly adaptive and personalized therapies, it will become imperative that we build devices that can accurately sense, predict, and react to a patient’s dynamic physiological state, such as their individual sleep cycles. Similarly, our role in “closing this loop” will increase as technology and clinical trials expand. With the advent of increasingly sophisticated therapies, the importance of maintaining an awareness of the patient experience, their safety, and their lifelong access and dependence will become more critically important. The recent emergence of open source platforms, as well as historical lessons learned from the development of the automated external defibrillator (AED), will help us to prepare for and move toward robust, reliable, and patient-centered technology.

### Balancing predictive and reactive control in bioelectronic systems: towards “circadian-aware” neuromodulation for neurological conditions

Neuromodulation therapies, such as DBS, are established treatments for conditions including movement disorders, chronic pain, and epilepsy, and are being explored for application to disorders such as depression, dementia, and OCD. Traditionally, DBS delivers continuous stimulation, or in more advanced systems, adaptive stimulation based on physiological feedback. However, clinical settings are typically optimized during daytime consultations and then applied uniformly across a 24-h cycle. This simplified approach may overlook the role of circadian rhythms and sleep in human physiology.

Sleep plays essential cognitive and physiological roles in the human, and its disruption is both a symptom and potential driver of many neurological and psychiatric conditions. DBS often targets brain regions closely linked with arousal and sleep regulation, meaning that stimulation patterns optimized during wakefulness may have unintended effects during sleep. Furthermore, neural biomarkers used for adaptive stimulation may shift significantly between wake and sleep states, and this challenges the “one size fits all” paradigm.

Physiology itself balances predictive and reactive strategies in an attempt to regulate complex interdependent systems. Circadian rhythms exemplify predictive control, anticipating regular changes, while homeostatic feedback provides reactive adjustments. By mimicking this natural predictive-reactive architecture, next-generation neuromodulation devices have the potential to deliver therapies that are more aligned with biological processes.

Recent evidence from long-term intracranial recordings in PD, major depressive disorder, and epilepsy highlight the importance of this approach. Future research systems should embody predictive-reactive control, integrating circadian awareness with responsive modulation. By aligning therapy with daily (and other) cycles, the aim of this approach is to improve symptom control, reduce sleep disruption, and ultimately break harmful feedback loops between neurological symptoms and disturbed physiology.

### Lessons from the development of the first bystander-usable automated external defibrillator

Physiological closed-loop (PLCL) control systems are increasingly central to the development of advanced neuromodulation therapies. Many of the foundational principles for their safe and effective deployment, however, were first demonstrated in other medical technologies. This presentation examines the first bystander-usable automated external defibrillator (AED) as a widely deployed PLCL system and draws lessons relevant to the design of adaptive deep brain stimulation (DBS).

This AED incorporated design offered elements later formalized in IEC 60601-1-10, the international standard for PLCL safety. Its architecture included robust sensing with an independent data quality channel, high-performance algorithms capable of rapid therapy decisions under noisy physiological conditions, and it safeguards to ensure that the therapy delivery did not compromise sensing. The use of independent hardware limits constrains the maximum therapy energy, while built-in self-tests ensures readiness before each use.

Through worldwide adoption, this system demonstrated how rigorous engineering of physiological feedback mechanisms can yield reliability, safety, and public acceptance in life-critical applications. DBS systems face analogous challenges, ranging from artifact rejection to robust sensing, and also add layered safety controls. This is particularly relevant to current-generation DBS technology, as devices have real-time sensing capabilities and adaptive sensing paradigms based on sensing data. By analyzing lessons from the first widely deployed bystander AED, we can inform the development of next-generation neuromodulation systems that are adaptive, resilient, and durable in clinical practice.

### Forking open-source platforms for your taste

Development of new neuromodulation technologies has traditionally been confined to proprietary, closed-source frameworks. Applying Open-source (OS) principles for neuromodulation device development has the potential to decrease the time and cost, especially during the initial stage after establishing the startup and entering a stage called the valley of death. Several OS medical device projects have been initiated through community collaboration and federal grant funding:

1. CCC: an engineering company in Uruguay that developed IPGS for 20 + US neuromodulation startups
2. OpenMind: a software suite for closing the loop in commercial DBS devices, such as Medtronic’s Summit RC + S and other commercial neuromodulation devices, funded by the NIH Brain Initiative
3. OpenNerve: a clinical-grade implantable nerve stimulator, funded by the NIH SPARC

**Figure 7:**
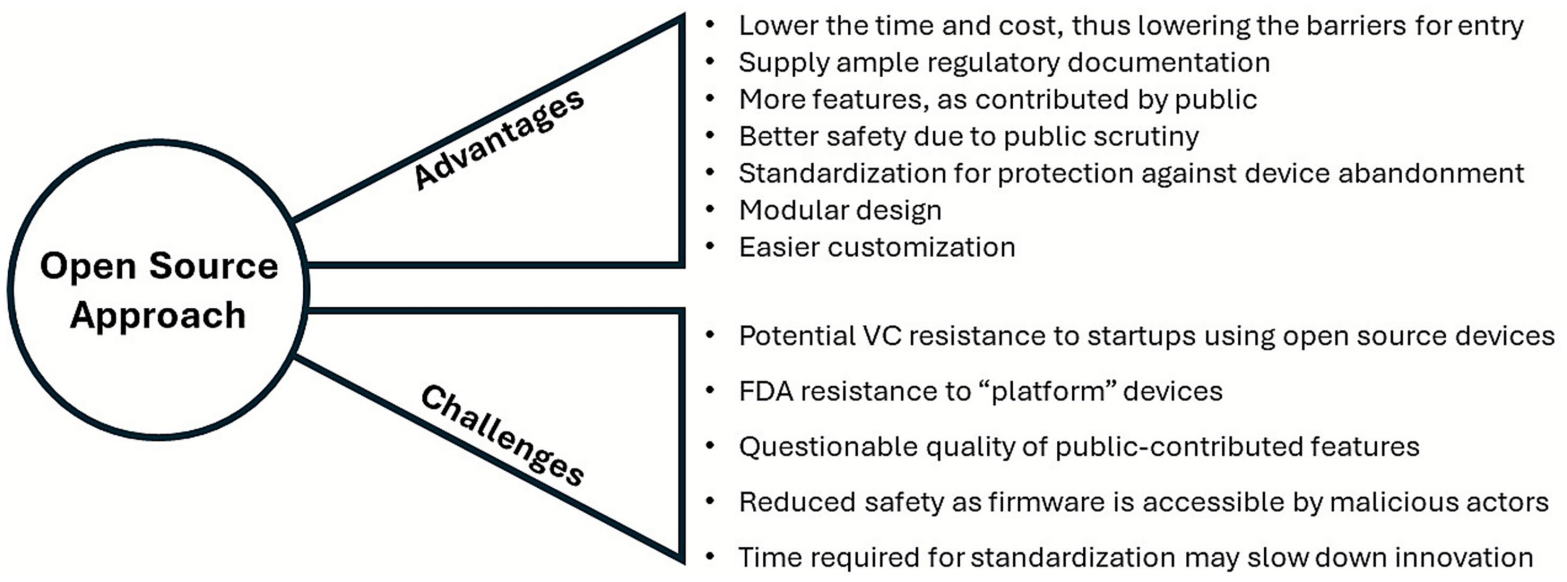
Advantages and challenges of following the open-source approach.

As shown in Figure 7, OS approach offers significant advantages, including lowering the time and cost, thus lowering the barriers for entry, supplying ample regulatory documentation, allowing for more features, as contributed by the public, and offering better safety due to public scrutiny, as well as standardization for protection against device abandonment, modular design, and easier customization. However, this approach also has distinct challenges. These include potential venture capital resistance for supporting startups that use open-sourced devices, FDA resistance to “platform” devices, questionable quality of public-contributed features, reduced safety as firmware is accessible by malicious actors, and increased time required for standardization that may slow down innovation.

To address this, startups can take advantage of a permissive license (Creative Commons CC-BY 4.0), which includes no exclusive licensing and no restriction for commercialization or patenting. Within these measures, startups can still develop and create intellectual property, including novel clinical indications and anatomical target, novel leads, and surgical deployment methods, as well as new stimulation parameters and algorithms.

**Figure 8:**
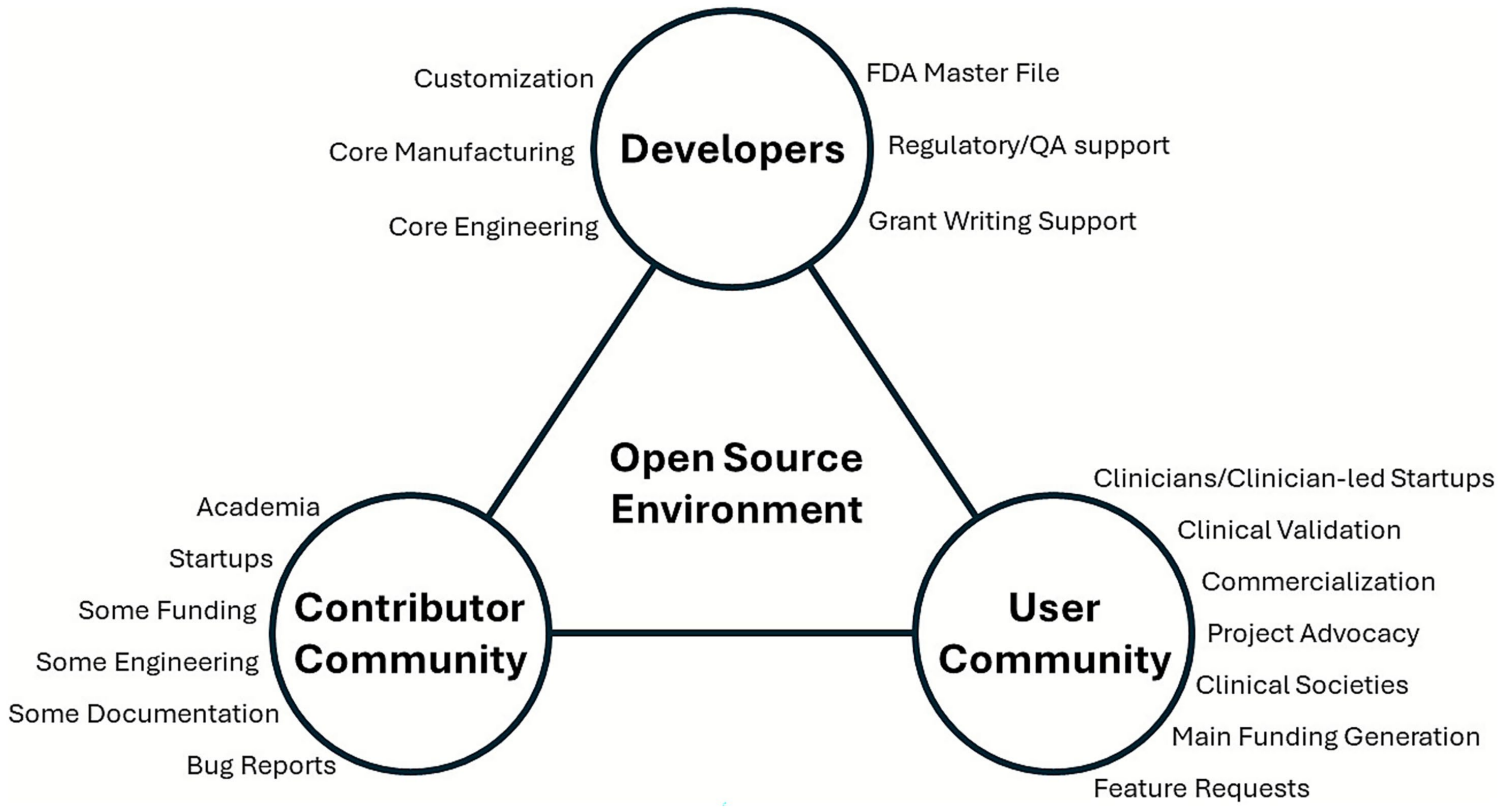
The three main groups that are vital for sustaining a technological environment that is conducive to open-source development.

In order to foster an OS community, it is important to create the ecosystem for sustaining an open-source project that includes three parties, as shown in Figure 8. First, there are the developers that include core engineering, core manufacturing, customization teams, regulatory and quality assurance support (including FDA Master File), and support for grant writing. Next, there is the contributor community, which includes academia and startups, some modes of funding, additional engineering, community documentation support, and bug reports. Finally, there is the user community, which includes clinicians and clinician-led startups, main funding generators, clinical validation and commercialization, project advocacy groups in clinical societies, and groups to provide feature requests.

## Physiology and closing the loop

Deep brain stimulation and neuromodulation in general has been shifting away from a static and open-loop model toward more dynamic, adaptive, biomarker-driven systems that account for and react to patients’ distinct physiological signatures and to address specific symptoms. This shift has facilitated the development of more tailored therapies for classical applications of DBS, such as motor control in PD, as well as the extension of DBS into new conditions, such as regulating tics in Tourette syndrome (TS). Recent clinical work has demonstrated the potential for personalized feedback to not only improve symptoms, but also to improve quality of life measures.

### Implementing adaptive DBS: from beta band dynamics to clinical practice

DBS is a very effective treatment for PD, although we do not consider the individual symptom fluctuations when applying chronic DBS. The aim of adaptive DBS is to adjust therapy with respect to the occurrence of individual motor symptoms in PD patients based on an identified feedback signal. Dr. Andrea Kuhn and her team propose that cortico-basal-ganglia motor network activity patterns can be attributed to distinct behavioral macro- and micro-states similar to other complex dynamic physiological processes (Signoret-Genest et al., 2023) and these patterns need to be evaluated as feedback signals for aDBS. Here, long-lasting macro-states would be influenced by disease progression, whereas rapid micro-states could represent medication or movement-induced acute network changes.

**Figure 9:**
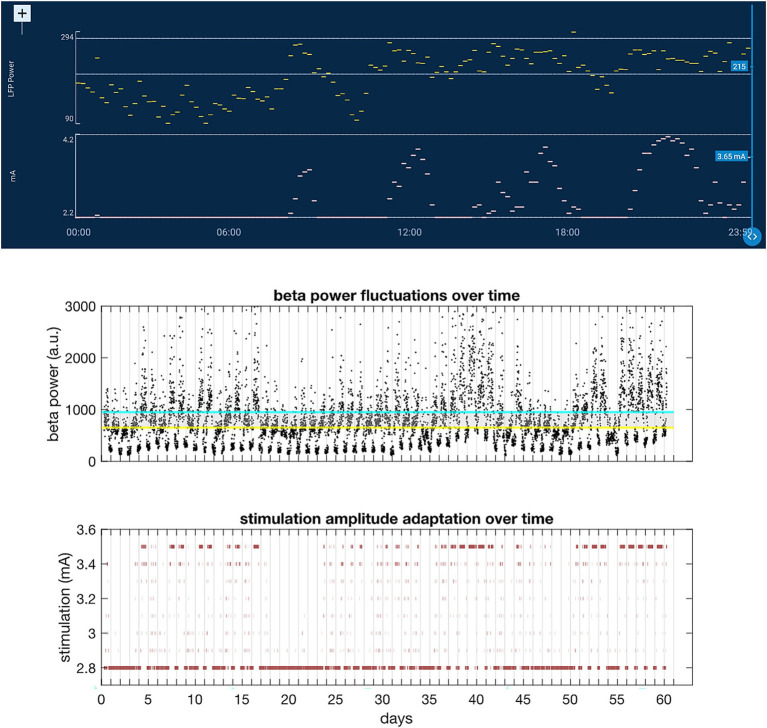
The top figure represents 24 h of timeline data with LFP power (upper trace, yellow line) and stimulation amplitude (lower trace, pink line). The bottom figure represents long-term beta power fluctuations over a 60-day time-period, which complicated threshold setting for aDBS. In the bottom figure, the blue and yellow lines represent the upper and lower beta threshold set for initial aDBS settings, respectively. Beta band fluctuations over longer time periods are shown in the plot that lead to maladjustment of stimulation amplitude if outside set thresholds. The reason for these fluctuations is still unknown—they may relate to fluctuations of the aperiodic component, but this needs further study.

The best studied biomarker so far in PD is STN beta band activity that correlates with the severity of motor symptoms, and beta band suppression during DBS reliably correlates with motor improvement (Feldmann et al., 2021) (Figure 9). In order to explore beta activity as a feedback signal for aDBS, the team showed that subthalamic beta power stabilizes only after about four weeks post-surgery due to the stun effect and remains relatively stable over several months (Feldmann et al., 2021; Behnke et al., 2025). Further, they described circadian fluctuations with beta band reduction during sleep (van Reekum et al., 2000) and these should be considered when searching for an aDBS parameter setting.

Adaptive DBS using beta band as a feedback signal has been available in clinical practice since January 2025. In the first aDBS study in 8 patients using EMA questionnaires as an outcome measure, a significant increase in overall well-being during adaptive DBS as compared to chronic DBS was shown, and a trend arose forecasting improvement of general movement. Six out of eight patients remained on chronic aDBS. Importantly, aDBS settings needed several sessions for adjustment (mean *n* = 7) and the mean stimulation amplitude range was rather small (0.6 mA). Dr. Kuhn’s team proposes the following guideline for switching from chronic to adaptive DBS in PD patients as a 3 steps procedure including (i) a preparation visit to select the beta peak and start timeline acquisition; (ii) a set-up visit to evaluate timeline and to set LFP thresholds and stimulation amplitudes; and (iii) optimization visits for adjustment of parameters (Van Rheede et al., 2022). Although there are still pitfalls and challenges associated with adaptive DBS, such as unstable beta thresholds and movement artifacts that may interfere with aDBS programming, the initial results are very encouraging, with patients benefiting from adaptive stimulation over chronic DBS.

### Defining targets for detecting and suppressing tics in Tourette syndrome

The human brain consists of numerous networks distributed over space and connected over time to orchestrate meaningful interaction with the external world. Neurological disorders interrupt this interaction, and in some cases, they lead to tic disorders. Bidirectional neurostimulators facilitate biomarker identification for symptoms, and responsive stimulation overcomes many downsides of continuous DBS therapy as a method to possibly regain control of movement. Dr. Aysegul Gunduz and the Brain Mapping Laboratory at the University of Florida have shown the feasibility and safety of bidirectional therapy, i.e., closed-loop stimulation, for TS. They are now investigating the efficacy of closed-loop DBS targeting multiple nuclei, including the centromedian (CM) nucleus of the thalamus and the anterior globus pallidus interna (aGPi). The trajectory forward of this work involves not only refining DBS parameters for tics, but also exploring the impact of CM and aGPi stimulation on TS-associated psychiatric comorbidities by tailoring therapies to individual patient profiles. They also aim to account for potential variations in response due to psychiatric comorbidities and to enhance treatment outcomes and improve the quality of life for TS patients. In their current NIH Brain Initiative study, Dr. Gunduz’s team has implanted bilateral CM DBS plus bilateral aGPi DBS and they are exploring detection and suppression paradigms across the neural network implicated in the generation of tics.

### Adaptive DBS for gait and freezing of gait in Parkinson’s disease: insights from ADAPT-PD

Dr. Helen Bronte-Stewart’s laboratory applied an aDBS paradigm for gait impairment and Freezing of Gait (GI&FOG) in PD using a beta band burst duration (BBBD) classifier. BBBDs are longer during normal gait in people with GI&FOG when compared to non-freezers and are even longer during periods of FOG within freezers (Anidi et al., 2018). In this study (UH3NS107709), they demonstrated the safety, tolerability and efficacy of aDBS for GI&FOG driven by personalized BBBD and by using a single threshold control policy (Wilkins et al., 2025). Adjusting DBS amplitude based on BBBD at 140 Hz resulted in improvement in GI&FOG in freezers and in MDS-UPDRS III and subscores. The results were equivalent to cDBS (continuous open loop). At 60 Hz aDBS and cDBS improved GI&FOG in freezers and was associated with no improvement in MDS-UPDRS III, bradykinesia or tremor compared to OFF DBS. MDS-UPDRS III and subscores were worse ON 60 Hz aDBS and cDBS when compared to cDBS (140 Hz).

The Adaptive DBS using Personalized Therapy in PD (ADAPT-PD) study: primary outcome and conclusions of the international multicenter pivotal trial were published separately (Bronte-Stewart et al., 2025). The unblinded comparisons among cDBS, Single Threshold (ST)-aDBS, Dual Threshold (DT)-aDBS, as defined by an upper and lower bound to guide stimulation amplitude, and the mode selected for the longterm phase (selected-aDBS) demonstrated significant and clinically meaningful increases in on time and decreases in off time during DT-aDBS and selected-aDBS when compared to cDBS. Participants were blinded to the mode of aDBS and selected their preferred mode when blinded however they were unblinded to cDBS versus aDBS. The average on time ON cDBS was similar to that published in a previous large multicenter trials of STN DBS (Weaver, 2009; Schuepbach et al., 2013).

The primary end-point from the recent ADAPT-PD study was met in terms of performance in both the ST (79% of participants) and DT (94% of participants) via *post hoc* thresholds, with no significant differences detected between groups (*p* = 0.51) (Bronte-Stewart et al., 2025). The secondary endpoint pertaining to total electrical energy delivered (TEED) was found to be reduced by 15% (mean) during both ST-aDBS and DT-aDBS when compared to cDBS (*p* = 0.01). No persistent adverse effects related to stimulation occurred, and exploratory analysis suggested that DT-aDBS may offer improvement in on-time with fewer notable dyskinesias.

## A roadmap for genetics and neuromodulation

As genetic testing is becoming more accessible and routine, spearheaded by initiatives like PD GENEration, the therapeutic landscape of DBS is shifting from a one-size-fits-all approach to one that personalizes therapy possibly in the future based on genetics.

### Targeted genetics and precision deep brain stimulation for movement disorders

Precision medicine centers on the ability to provide the right treatment for the right patient at the right time. DBS for movement disorders follows the precision medicine path by bringing an individualized approach that is tailored to the needs and symptoms of patients (Cavallieri et al., 2024). New to the movement disorders playing field is the advent of widespread genetic testing. By linking genetic status to DBS outcomes, genetic testing can provide insights into the nature of a particular movement disorder, can inform clinical decision-making, and may even validate the diagnosis for a few individuals (Trevisan et al., 2024). For example, do patients with certain specific genetic variants, e.g., in the *GBA1* gene, fare better or worse with DBS? Should a patient’s genetic status suggest earlier vs. later intervention in order to maximize the benefit of DBS? To overcome that knowledge deficit and reap the potential impact, genetic testing should be included in the routine work-up of patients considering DBS (Gan-Or, 2024). Genetic testing is readily available, and, in the case of those with a diagnosis of Parkinson’s disease, is provided at no cost through the PD GENEration study sponsored by the Parkinson’s Foundation. While data accrue to answer key questions around DBS and genetics, there remains a fundamental benefit and recognized personal utility, if not clinical utility, for patients undergoing genetic testing and receiving the return of their results (Oas et al., 2024). In short, genetic testing provides an opportunity to bring benefit to patients and to deliver a new level of refinement in the precision application of DBS to movement disorders.

### Can PDGene help us improve DBS planning?

DBS has demonstrated robust motor benefits in PD; however, outcomes for non-motor symptoms may be more heterogeneous. Evidence suggests the potential negative effects of DBS on cognition and apathy and these concerns are amplified in patients with monogenic forms of PD, particularly carriers of *GBA* mutations. Although clinically indistinguishable from idiopathic PD, *GBA*-associated PD is characterized by faster progression and heightened vulnerability to cognitive impairment. Additionally, evidence from studies following individuals with PD-GBA who have undergone STN DBS, has suggested that this population may have an accelerated cognitive decline following surgery.

To date, most investigations have been retrospective, narrowly focused on cognitive outcomes, and often limited by suboptimal control groups. Moreover, comparable data on globus pallidus interna (GPi) DBS, a target frequently considered more conservative regarding cognition, remain sparse.

The PD GENEration study represents the largest genetic initiative in PD, offering free testing and counseling for the seven most common PD-related mutations across the U.S. sites and also globally. To date, over 27,000 participants have been enrolled, with positivity rates of ~12%, of which 58% are *GBA* carriers. This infrastructure provides a unique opportunity to integrate genetics beyond GBA status and DBS, to advance precision medicine in PD.

Future research should investigate variability in DBS response across multiple monogenic forms of PD, while incorporating broader outcome measures, including patient-reported experiences. While current evidence supports the use of genetic testing to inform preoperative counseling for DBS, large-scale, prospective studies are critically needed to clarify risk profiles and to optimize patient-centered decision making.

### Genetic testing in deep brain stimulation for dystonia: opportunities and challenges

DBS is an effective therapy for dystonia; however, outcomes remain heterogeneous. Etiology and clinical phenotype are strong predictors of response, yet dystonia itself represents a constellation of disorders with very diverse biological substrates. Increasingly, genetic testing offers an additional layer of diagnostic certainty, prognostic value, and mechanistic insight. Established isolated dystonia genes such as *TOR1A* and *THAP1* illustrate a spectrum of DBS responsiveness in isolated dystonia genes, while more recently identified genes, including *KMT2B* and *VPS16*, highlight both overlapping phenotypes and important differences in response profiles. Beyond prediction, genetics also reveals circuit-level distinctions, as shown by genotype-specific electrophysiologic signatures and the emergence of episignatures in *KMT2B*, representing the first dystonia-related biomarker, with potential relevance for DBS candidacy.

Despite these advances, challenges remain. Genetic testing is increasingly available; however, issues of cost, counseling, and coverage persist, especially in adult-onset focal dystonia. Selective reporting of positive outcomes also risks biasing the literature. Systematic and transparent reporting across monogenic dystonias will be essential to build an evidence base that can inform clinical decision-making. Ultimately, integrating genetics into the DBS evaluation facilitates not only refinement of candidacy and prognostication but also the movement toward a framework of precision neuromodulation applied to dystonia.

### Personalized neuromodulation: the role of genetics in deep brain stimulation for movement disorders

Despite the significant genetic contributions of single variants in *GBA1*- and *LRRK2*-linked PD, there remains substantial clinical and pathological heterogeneity. Growing evidence supports the influence of additional genetic and environmental factors on disease onset and progression, which may also possibly impact treatment outcomes.

A critical issue arises concerning whether PD patients with *GBA1* variants, due to their potentially increased risk of dementia following DBS (Pal et al., 2023; Fernández-Vidal et al., 2024; Avenali et al., 2024), should undergo the procedure. This raises a discussion on pre-surgical genetic testing to enable risk stratification and to facilitate shared decision-making. However, a better understanding of how individual *GBA1* variants interact with other influencing factors is necessary to inform treatment decisions. Moreover, the role of biomarkers, such as *α*-synuclein status [e.g., quantification of α-synuclein seeding amplification assays and α-synuclein PET imaging (which is not yet available)] and coexisting pathologies, merits further study to help guide therapeutic strategies.

Another essential task is the systematic longitudinal monitoring of non-surgically treated PD patients with *GBA1* variants, as well as patients receiving alternative follow-up therapies, such as focused ultrasound and pump therapies. These studies are vital for identifying predictors of dementia development across different treatment regimens. In addition, careful consideration will be required to evaluate whether the cognitive risks associated with DBS outweigh their substantial benefits on motor and other non-motor symptoms, as well as their overall quality of life. Larger cohorts, such as those from PDGeneration (Cook et al., 2024) and the Global Parkinson’s Genetic Program (GP2), although not specifically designed to evaluate DBS outcomes, could provide valuable insights into the relationship between genetic factors and DBS responses in PD.

In the context of other genetic models, X-linked dystonia parkinsonism (XDP) offers a relevant example of how genetic modifiers may influence disease progression. XDP is marked by significant striatal neurodegeneration detectable via MRI, with quantifiable changes in brain structure. Genetic factors may predict disease onset during the prodromal phase and are associated with the extent of striatal atrophy. DBS of the globus pallidus internus (GPi) has shown excellent results, particularly when neurodegeneration is less advanced (Brüggemann et al., 2019), supporting the potential for early intervention. In contrast, DBS outcomes in Huntington’s disease remain less consistent, with improvements in hyperkinesia often overshadowed by disease progression.

## Conclusion

The 13th annual DBS Think Tank showcased cutting-edge advancements in neurostimulation and, over the course of three days, sparked multiple significant and engaging discussions regarding neuroethics, technology, industry, and possible paths forward. A primary focus of the discussion was the ongoing paradigm shift from open-loop stimulation towards dynamic, biomarker-driven therapies. The meeting covered an overarching theme of increasingly tailored and patient-specific forms of neuromodulation therapy. The transition has been powered by recent breakthroughs in aDBS using real-time neural biomarkers to treat motor symptoms in PD, as well as by non-motor symptoms, such as mood and impacts on decision-making. Concurrently, the concept of connectomics-based DBS has begun to break into clinical practice, allowing researchers and physicians to move beyond traditional anatomical targeting towards more robust and sophisticated frameworks. This newer direction considers patient-specific variables, as well as distributed targets beyond those immediately available, paving the way for “adaptive circuit targeting” and the possibility of more flexible network-based treatments.

The drive toward personalization has sparked the integration of genetics into clinical practice. This may, in some cases, refine patient selection, the specificity of the therapy applied, and prognostication for conditions like PD and dystonia.

Industry presented key technological innovations ranging from high-density directional leads and predictive algorithms to digital health platforms enabling increased access via remote patient care. As the capabilities of these neurotechnology systems expand, so will the associated societal and ethical impacts. Critical discussions arose regarding increasingly pressing issues within this frontier, including continuity of care and efforts to address “abandonware,” as well as a need to combat public stigma, which were all hot topics. Additionally, the Living Brain Project provided an opportunity for a meaningful dialogue on appropriate methods for consenting and for the performance of brain biopsies during unrelated elective procedures. Genetics, connectomics, and biosensing, are converging into an increasingly patient-specific paradigm and hold promise to transform DBS from a localized tool to more personalized and precise circuit-based intervention.

**Figure 10:**
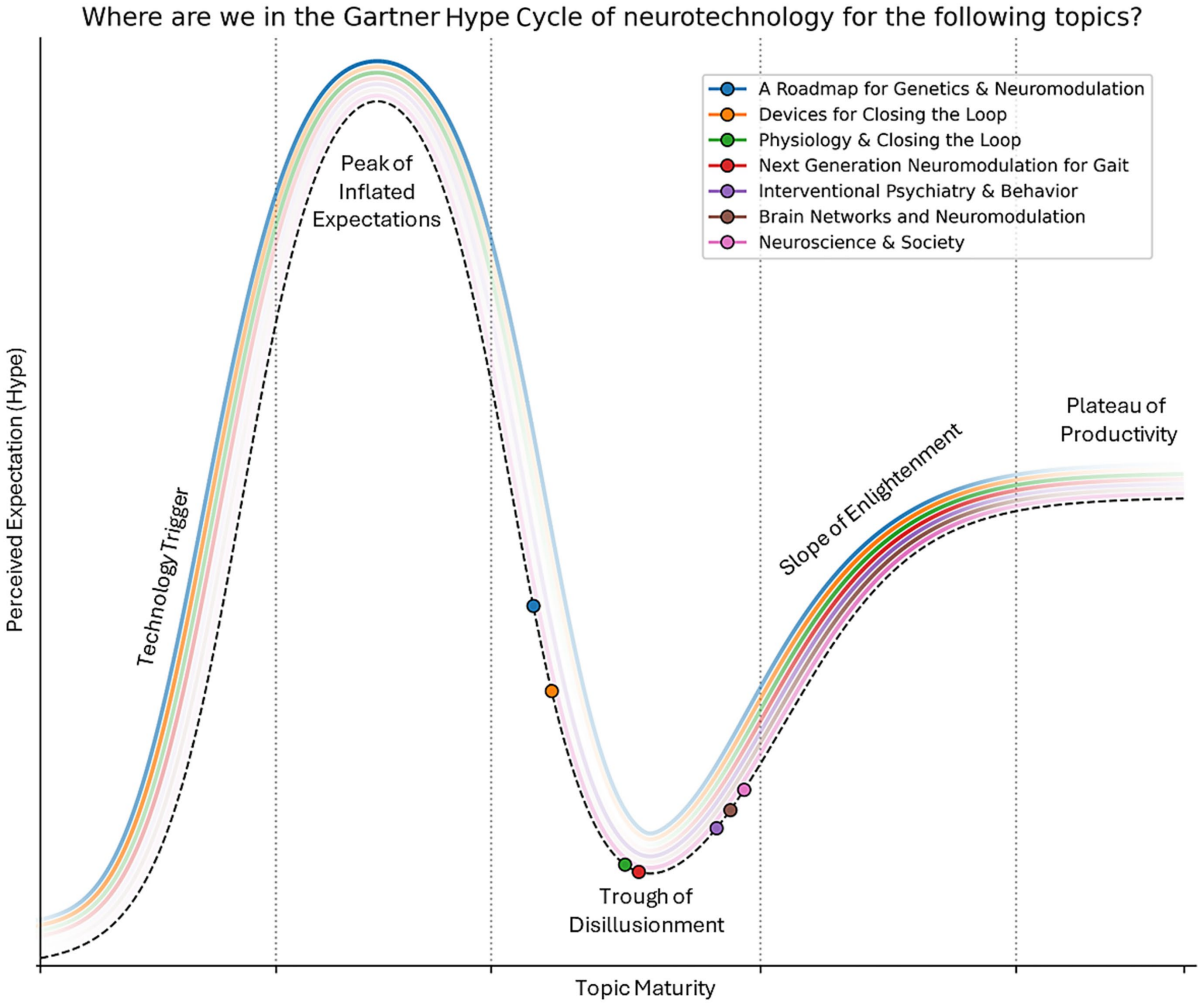
Distributed votes in response to the question “Where are we in the Gartner Hype Cycle of neurotechnology…” for each topic discussed at DBS Think Tank XIII projected over the standard Gartner hype cycle curve (dashed line). For each topic, the density of votes at a given point is portrayed as the opacity of their respective color-coded lines and the mean location for each topic is shown by their respective color-coded dot on the Gartner hype cycle curve.

The growth and subsequent evolution of perceived expectations specific to each topic were captured within the responses to our post-session survey (Figure 10). The highest density of votes for each topic favored the “Slope of Enlightenment” phase of the standard Gartner Hype Cycle, meaning the attendees generally felt that each technology has matured to the point that its popularity is approaching its true stable practical value. This also suggests that each topic has successfully escaped the “Trough of Disillusionment,” the crash in interest that often occurs once initial novelty wanes from a topic— a state that often limits widespread adoption. However, many of the remaining votes alternatively categorized the topics into earlier phases of their Gartner Hype Cycle, mainly their “Peak of Inflated Expectations” and “Technology Trigger” phases. These both describe the initial wave of interest and popularity that follows breakthroughs and novel technologies with readily apparent potential. While together this split situated the mean vote for each topic within the “Trough of Disillusionment,” a location with few outright votes, the overall distribution for each describes optimistic heavily tailed distributions, with strong forward momentum towards the “Slope of Enlightenment,” which associates with the stable, lasting, and broad applications of these technologies within an appropriate context.

The path forward demands an unwavering dedication to navigate this complex landscape, but if done carefully, it promises unprecedented therapeutic benefit for all of our patients.
